# A retrospective study of HIV and hepatitis B co-infection at a tertiary hospital in the Western Cape province of South Africa

**DOI:** 10.4102/sajhivmed.v27i1.1847

**Published:** 2026-10-06

**Authors:** Alida M. Hugo, Tamsin Lovelock, Jantjie J. Taljaard

**Affiliations:** 1 Division of Infectious Diseases, Department of Medicine, Faculty of Health and Sciences, Stellenbosch University, Cape Town, South Africa

**Keywords:** HIV/HBV coinfection, chronic hepatitis B, HIV infection, South Africa, sub-Saharan Africa, liver fibrosis, retention in care, outcome

## Abstract

**Background:**

HIV and hepatitis B virus (HBV) coinfection is a clinical concern because of shared transmission routes and accelerated liver disease progression. Data from sub-Saharan Africa (SSA) remain limited.

**Objectives:**

To describe the clinical, laboratory, radiological, and outcome characteristics of adults with chronic HIV/HBV coinfection attending a tertiary hospital in South Africa.

**Method:**

A retrospective observational study of adults with HIV and chronic HBV infection attending an infectious diseases clinic between 01 January 2019 and 31 October 2024. Chronic HBV was defined as hepatitis B surface antigen positivity for at least 6 months. Eligible patients had at least two clinic visits. Data were extracted from clinical, laboratory, imaging, and pharmacy records.

**Results:**

Of the 179 patients screened, 140 met inclusion criteria. The cohort had a female predominance of 51.4% and a median age of 42 years; most were diagnosed through routine HIV care. The median CD4 count was 214 cells/μL, and many had advanced immunosuppression. Most were receiving antiretroviral therapy (ART), with 78.8% on tenofovir (TDF)-based regimens. HIV and HBV viral load (VL) was low, and the median HBV VL was 81 IU/mL. Liver assessment showed that 36.3% had an abnormal Aspartate Aminotransferase to Platelet Ratio Index score and 40.2% had an elevated liver stiffness measurement (> 7 kPa) suggestive either of significant fibrosis or cirrhosis. Fifteen patients died, 3 of whom died from HBV-related causes. Retention in care was poor, with only one-third remaining in follow-up.

**Conclusion:**

Chronic HBV remains an important comorbidity among people living with HIV in South Africa, particularly in those born before widespread vaccination. Despite high ART coverage, important gaps remain in fibrosis assessment and retention in care. Strengthening prevention, vaccination, diagnostic access, and continuity of care is essential to reduce HBV-related morbidity and mortality.

**What this study adds:** Limited data on HIV/HBV coinfection in SSA hinder efforts to improve HBV care. Studying affected patients can clarify outcomes in the ART era, inform public health interventions, and support progress toward the WHO 2030 viral hepatitis targets.

## Introduction

Viral hepatitis remains a major global health challenge. The 2026 WHO Global Hepatitis Report estimated that 1.1 million deaths in 2024 were because of hepatitis B virus (HBV)-related cirrhosis and liver cancer, a 17% increase since 2015. Approximately 75% of these deaths occurred in the WHO African and Western Pacific regions, and South Africa is among the top 10 countries contributing to the global burden. In 2024, 240 million people were living with chronic HBV and 0.9 million were newly infected. The WHO African region accounted for 68% of new infections, with a substantial proportion of chronic disease concentrated in sub-Saharan Africa (SSA). Although HBV is vaccine preventable, gaps in vaccine availability, coverage, and uptake persist in many low- and middle-income countries.^1,2^

Chronic HBV is defined by persistence of hepatitis B surface antigen (HBsAg) for at least 6 months. Chronicity is strongly age dependent, occurring in up to 95% of perinatal or early childhood infections, but in only approximately 5% of adult infections among people without HIV.^3,4^

HIV and HBV share blood-borne transmission routes, making coinfection clinically important especially with regard to their synergistic negative effects on liver disease. Globally, an estimated 2.7 million people are coinfected, with SSA contributing 69% of cases. In developing countries, HBV is primarily acquired, either vertically or horizontally, in early childhood, whereas HIV is mainly acquired sexually in adulthood; in developed countries, both infections are acquired mostly through unprotected sex or contaminated injection equipment.^4,5,6,7,8,9^

Evidence from high-income settings shows that HIV accelerates HBV progression through higher HBV DNA levels, reduced hepatitis B e-antigen (HBeAg) clearance, and increased risks of cirrhosis, hepatocellular carcinoma (HCC), and liver-related mortality. HIV also increases risk of chronicity after acute HBV exposure, particular at lower CD4 T-cell counts.^10,11,12,13^

Data on HIV/HBV coinfection in SSA remain limited. Matthews et al.^9^ report that HIV/HBV co-infected patients in Africa are predominantly male and older. Msomi et al.^8^ describe a small cohort of patients with co-infection in South-Africa, finding that advanced HIV (CD4 < 200 cells/μL) was an independent risk factor for persistent HBV viraemia. Studies from Kenya and Tanzania similarly report male predominance and widespread immunosuppression, as well as raised alanine transaminase (ALT) and lower platelet counts.^14,15^

Expanded antiretroviral therapy (ART) access has improved survival among people living with HIV, but liver disease has become a major cause of morbidity and mortality. In South Africa, there is a 15% – 25% chance of dying from HBV-related liver disease in HBV-mono-infected individuals.^4,6,16,17,18^ HIV may increase HCC risk up to six-fold compared to those not living with HIV. In South Africa, the 5-year mean survival ranges between 25% and 60%, while non-resectable tumours have a mean survival of 5 months.^16,17,19,20^

All people with HIV/HBV co-infection should receive tenofovir (TDF)-based ART with lamivudine (3TC) or emtricitabine (FTC), regardless of disease stage or HBV DNA level. This achieves high rates of HBV DNA suppression (90%), HBeAg loss (46%) and HBsAg loss (12%). It also reduces progression to cirrhosis.^2,3,16,21^

This study describes the clinical, laboratory, and radiological characteristics, and treatment patterns and outcomes, of adults with HIV/HBV coinfection attending a tertiary infectious diseases clinic (IDC) in South Africa.

## Research methods and design

### Study design and population

This retrospective observational study was conducted at Tygerberg Hospital, a tertiary academic hospital in Cape Town, located in the Western Cape province of South Africa. Adult patients with HIV and chronic HBV infection who attended the IDC for their first visit between 01 January 2019 and 31 October 2024 were screened. Inclusion criteria were confirmed chronic HBV infection (defined as persistent HBsAg positivity for ≥ 6 months) and at least two IDC visits. Patients with insufficient evidence of chronic HBV, only one visit during the study period, or age < 18 years were excluded. Data were obtained from clinical records, including physician notes, laboratory results, imaging reports, and pharmacy records.

### Statistical analysis

Data were analysed descriptively. Categorical variables were summarised using frequencies and percentages. Numerical variables were grouped into categories and summarised using frequencies, percentages and mean or median values.

### Ethical considerations

Ethical clearance to conduct this study was obtained from the University of Stellenbosch Health Research Ethics Committee (HREC) (reference no: HREC S25/03/038). A waiver of consent was granted as this was a retrospective descriptive study analysing patient folder data which was deidentified. Approval was also sought and granted from hospital management at the study site. All data were managed in compliance with ethical guidelines to ensure patient confidentiality. The patient information and data were stored securely on the primary investigator’s password-protected computer.

## Results

Between January 2019 and October 2024, 179 patients presented to IDC with HBV and HIV co-infection, of whom 140 met the inclusion criteria.

Table 1 summarises the baseline clinical characteristics of the study population. There was a slight female predominance in the cohort. The median age was 42 years. Most individuals were middle-aged adults between 30 years and 49 years (*n* = 101, 72.1%).

**TABLE 1:** Study population demographic, disease and treatment characteristics at Tygerberg Hospital between January 2019 and October 2024.

Description or category	*n*	%	Median
**Gender**	140	-	-
Male	68	48.6	-
Female	72	51.4	-
**Age (years)**	140	-	42
18–29	12	8.5	-
30–49	101	72.1	-
≥ 50	27	19.2	-
**CD4 count (cells/μL)**	127	-	214
< 200	62	48.8	-
200–500	37	29.1	-
≥ 500	28	22.1	-
**Reason for HBV testing**	140	-	-
Routine or integrated HIV care	102	72.9	-
Clinical suspicion of HBV disease	37	26.4	-
Unknown	1	0.7	-
**On ART**	140	-	-
Yes	123	87.9	-
No	17	12.1	-
**On TDF**	97	78.8	-
**HIV viral load suppressed**	140	-	-
Yes	82	58.5	-
No	36	25.7	-
Unknown	22	15.8	-

HBV, hepatitis B virus; ART, antiretroviral therapy; TDF, tenofovir.

Most patients were first tested for HBV as part of routine HIV care (*n* = 102, 72.9%); 26% (*n* = 37) were tested because of clinical suspicion of hepatitis and one case had an unknown indication.

The median CD4 count was 214 cells/μL, and nearly half of the cohort had advanced immunosuppression with a CD4 count < 200 cells/μL (*n* = 62, 48.8%). Most patients (87.9%) were already established on ART at the time of presentation; of whom, 78.8% were receiving a TDF-containing regimen. Of those on ART, only 58.5% had a suppressed HIV viral load on presentation.

Table 2 outlines the HBV-related laboratory parameters. The majority had a low HBV viral load (< 2000 IU/mL; *n* = 87, 66%), while 22.7% (*n* = 30) had a markedly elevated viral load exceeding 200 000 IU/mL. Hepatitis B e-antigen status was incompletely documented, with 38 patients (27%) lacking recorded results; among those with known status, 42 (30%) were HBeAg-positive.

**TABLE 2:** Laboratory results and calculated aspartate aminotransferase to platelet ratio index score at Tygerberg Hospital between January 2019 and October 2024.

Description or category	*n*	%	Median
**HBV DNA VL (IU/mL)**	132	-	81
< 2000	87	66.0	-
2000 – 20 000	10	7.6	-
20 000 – 200 000	5	3.7	-
> 200 000	30	22.7	-
**HBeAg**	140	-	N/A
Positive	42	30.0	-
Negative	60	43.0	-
Unknown	38	27.0	-
**ALT (U/L)**	139	-	27
< 40	98	70.5	-
40–80	23	16.5	-
> 80	18	13.0	-
**AST (U/L)**	127	-	36
< 40	74	58.3	-
40–80	36	28.3	-
> 80	17	13.4	-
**APRI score**	113	-	0.3
< 0.5	72	63.7	-
0.5–1.0	25	22.1	-
> 1.0	16	14.2	-

N/A, not applicable; APRI, Aspartate Aminotransferase to Platelet Ratio Index; HBV DNA VL, Hepatitis B DNA viral load; HepBeAg, Hepatitis B e-Antigen; ALT, Alanine transaminase; AST, Aspartate aminotransferase.

The median hepatic enzyme values were 27 U/L for ALT and 36 U/L for aspartate aminotransferase (AST). However, nearly one-third of participants (29.5%) had an ALT > 40 U/L, and 41.7% had an AST > 40 U/L.

The AST-to-platelet ratio index (APRI) was < 0.5 in approximately two-thirds of the cohort (*n* = 72, 63.7%); 22.1% had an APRI score suggestive of liver fibrosis and 14.2% had a score suggestive of cirrhosis. These individuals required further assessment with additional investigations such as transient elastography (TE) or abdominal ultrasound.^2^

Table 3 shows the imaging results. Within the cohort, 117 of the 140 patients had a documented TE measurement. The median liver stiffness measurement (LSM) for these patients was 6.2 kPa, but 27.4% had an LSM of 7 kPa or greater, suggesting liver fibrosis, and 12.8% had a markedly abnormal LSM of greater than 12.5 kPa, suggesting liver cirrhosis.^2^

**TABLE 3:** Transient elastography and abdominal ultrasound results at Tygerberg Hospital between January 2019 and October 2024.

Description or Category	*n*	%	Median
**Liver stiffness**	117	-	6.2
LSM < 7 kPa (F0–1)	70	59.8	-
LSM 7–12.5 kPa (F2–F3)	32	27.4	-
LSM > 12.5 kPa (F4)	15	12.8	-
**Liver ultrasound**	122	-	N/A
Normal	84	69.0	-
Abnormal	38	31.0	-
**Abnormal findings**	38	-	N/A
Hepatis steatosis	20	52.6	-
Hepatomegaly	5	13.2	-
Portal hypertension	7	18.4	-
Hepatocellular carcinoma	1	2.6	-
Hepatic lesion	4	10.6	-
Other	1	2.6	-

N/A, not applicable; LSM, Liver Stiffness Measurement; kPa, kilopascal.

Abdominal ultrasound was performed in 122 of the 140 patients as part of routine screening for hepatic steatosis, cirrhosis, portal hypertension, and HCC. Of these, 38 examinations (31%) were reported as abnormal. Hepatic steatosis was the most common abnormal finding, present in 20 patients (52.6%). Portal hypertension (*n* = 7, 18.4%) and hepatomegaly (*n* = 5, 13.2%) were the next most frequently observed features. Only one patient demonstrated convincing ultrasound features suggestive of HCC. A second individual was ultimately diagnosed with HCC on computed tomography; the preceding ultrasound had shown portal hypertension and a small, vague hypoechoic vascular lesion within the liver.

Three of the 15 patients who died during the study period were clearly attributable to HBV. Two deaths were caused by HCC and the third by liver failure. Most of the remaining deaths were related to HIV-associated opportunistic infections. Two additional patients died from end-stage renal failure. As shown in Table 4, the gender distribution among the deceased was nearly equal, with seven women and eight men. The median age at death was 41 years.

**TABLE 4:** Mortality result at Tygerberg Hospital between January 2019 and October 2024.

Cause of death (*N* = 15)	*n*
Hepatocellular carcinoma	2
Multi-drug-resistant tuberculosis	2
Cryptococcal meningitis	2
End-stage renal failure	2
Drug-sensitive tuberculosis	2
Acute gastroenteritis and hypokalaemia	1
Upper gastrointestinal bleed during gastroscopy	1
T-cell lymphoma	1
Liver failure	1
Listeria	1
**Distribution of deaths**	
Sex	
Male	8
Female	7
Median age of deaths	41

The outcome of the cohort is shown in Table 5. Only 33% (*n* = 47) were retained in care and more than half of patients were lost to follow-up at the study site, though evidence of healthcare contact at another facility was found in all but one of these patients.

**TABLE 5:** Patient outcome at Tygerberg Hospital between January 2019 and October 2024.

Description	*n*	%
Active in care	47	33.6
Known deceased	15	10.7
LTFU and known alive	77	55.0
LTFU and vital status unknown	1	0.7

LTFU, Lost to follow-up.

## Discussion

Hepatitis B remains endemic in South Africa despite the introduction of the HBV vaccine into the national Expanded Programme on Immunisation (EPI) in 1995.^22^ Individuals born before 1995, and therefore not eligible for universal infant vaccination, remain at increased risk of acquiring HBV and progressing to chronic infection if not vaccinated later in life. National coverage for the full three-dose vaccine series averaged 65.1% in 2025.^23^ This remains far below the WHO-recommended target of ≥ 90%.^24^

This descriptive study provides insight into the epidemiological, laboratory, and radiological characteristics of a cohort with HIV and HBV co-infection. It also highlights limitations in the initial clinical work-up of these patients, the frequent need for additional investigations, and the resulting strain placed on an already overburdened healthcare system.

A slight female predominance was observed in this cohort, aligning with national HIV prevalence data, which also shows higher HIV rates among women.^25^ In contrast, a recent HBV mono-infection study from South Africa reported a male predominance^26^ and several SSA studies of HIV/HBV co-infection similarly report higher rates among men.^14,15,27^ The female preponderance in our study may reflect improved HBV screening in people living with HIV or it may be attributed to the antenatal HBV screening performed to assess the need for administration of targeted HBV birth dose vaccine for infants.^18^

Most of the cohort was aged between 30 years and 49 years, which aligns with the known epidemiology of both HIV and HBV in South Africa.^27,28,29^ This age distribution also reflects the population born before the introduction of the HBV vaccine into the national immunisation programme, contributing to the higher prevalence of chronic HBV observed in this group.^26^ Individuals in this age range are typically economically active and often responsible for supporting dependants; consequently, illness or premature death in this group carries substantial social and economic implications. Among women, who comprised more than half of the cohort, this age group also corresponds to the reproductive years, underscoring the added risk of perinatal HBV transmission.^16^ In 2023, a policy document recommended that babies born to HBV-positive mothers receive HBV vaccination at birth and that HBV-negative pregnant women receive a full schedule of HBV vaccination.^30^

A large proportion of the cohort had advanced HIV, with CD4 counts < 200 cells/μL, similar to findings from studies in high-income countries.^13^ Severe immunosuppression likely contributed to the pattern of mortality observed: most deaths were attributable to HIV-related opportunistic infections, however 20% were clearly linked to HBV-related liver disease.

Of the cohort, 88% (123 of 140) were on ART at presentation, and 79% of these were receiving a TDF-containing regimen. This is reflected in the relatively low HBV viral load distribution, with 66% of patients demonstrating an HBV DNA VL < 2000 IU/mL. Those who have non-suppressed VL may be because of inadequate time on ART, non-adherence, or resistance. Only 58.8% of patients had a suppressed HIV VL on presentation. Standard first-line ART in South Africa includes TDF and national trends show improving ART uptake, with the Joint United Nations Programme on HIV/AIDS reporting an increase in ART coverage from 78% in 2021 to 83% in 2023.^28^

Most patients were tested for HBV as part of routine screening for people living with HIV and were asymptomatic of HBV infection. Despite this, a substantial proportion of patients required further investigation for possible liver fibrosis. The APRI score fell within the indeterminate range (0.5–1.5) for 26.6% of the cohort, and 40.2% had an elevated LSM (> 7 kPa). These findings present a clinical challenge: patients with inconclusive results require access to TE or specialist imaging, which is typically available only at secondary or tertiary centres. As previously noted, many affected individuals fall into the economically active age group and face practical barriers to attending repeated specialist appointments. When feeling well and already on ART, they may have limited understanding of the need for ongoing HBV monitoring. However, failing to attend follow-up places them at risk of presenting only once they develop complications of chronic HBV, at which point treatment options are limited and outcomes poor.^21^

Tertiary care follow-up may be less accessible than primary care follow-up, as demonstrated by the overall outcome of the study participants, with more than half of patients lost to follow-up at the study site but still documented to be in care at other sites. However, overall retention in care remains poor even for HIV mono-infection, with South African data from 2018 to 2023 demonstrating that only 61% of people initiating ART in public sector clinics remain in care at 12 months.^31^ A study from Mpumalanga in South Africa identified multiple barriers to retention in HIV care, including unclear communication from healthcare workers, perceived judgement or stigma, financial losses from missed work, and fear of attending clinics associated with HIV services.^29^ These factors likely contribute similarly to poor retention among individuals with HIV/HBV co-infection.

### Limitations

This study has several important limitations. The retrospective design relies on the accuracy and completeness of routinely collected clinical records, resulting in some missing or inconsistently documented data, particularly regarding HBeAg status and other laboratory variables. The study was unable to establish the relation of the HIV VL to when ART was initiated, time on ART and times of interruption. These limitations also influence the HBV VL data. As a single-centre study conducted at a specialist IDC in a tertiary hospital, referral bias is likely. Patients included in this cohort may represent a subset of HIV/HBV co-infection with more complex disease, abnormal investigations, or clinical features prompting referral, rather than the broader HIV/HBV co-infected population. The sample size is relatively small and the absence of systematic follow-up data for patients not retained in care limits assessment of long-term outcomes or progression to liver-related complications. The study period spans years in which HIV and HBV diagnostic and therapeutic guidelines have changed, which may have influenced patterns of screening, treatment initiation, and referral.

## Conclusion

This study highlights the ongoing burden of chronic hepatitis B among people living with HIV in South Africa, particularly within the economically active population born before the introduction of universal HBV vaccination. Positive aspects were the high ART coverage and widespread use of TDF-based regimens, with low HBV VL. Despite this, 20% of deaths in this cohort were directly related to HBV infection and a substantial proportion of patients required further evaluation for liver fibrosis, necessitating follow-up at a tertiary institution. Retention in care at the study site was poor, with implications for delayed diagnosis of liver disease and missed opportunities for prevention of long-term complications. These findings emphasise the need for strengthened HBV prevention strategies, including improved vaccination coverage, enhanced patient education, and health-system interventions that reduce barriers to continuity of care. Expanding access to appropriate diagnostic tools at lower levels of care and supporting long-term retention in care are essential to mitigate disease progression and reduce the future burden of HBV-related morbidity and mortality.
